# Essential Oils from Fructus *A. zerumbet* Protect Human Aortic Endothelial Cells from Apoptosis Induced by Ox-LDL *In Vitro*

**DOI:** 10.1155/2014/956824

**Published:** 2014-12-23

**Authors:** Yan Chen, Duo Li, Yini Xu, Yanyan Zhang, Ling Tao, Shouqiao Li, Yan Jiang, Xiangchun Shen

**Affiliations:** ^1^Department of Pharmacology of Materia Medica, Guiyang Medical University, Guiyang, Guizhou 550025, China; ^2^The Key Lab of Optimal Utilization of Natural Medicine Resources, Guiyang Medical University, Guiyang, Guizhou 550025, China

## Abstract

*Alpinia zerumbet* is a *miao* folk medicinal plant widely used in the Guizhou Province of southwest China that contains several bioactive constituents and possesses protective effects against cardiovascular diseases. In the present study, we evaluated the protective effect of essential oils derived from Fructus *Alpiniae zerumbet* (EOFAZ) on oxidized lowdensity-lipoprotein- (ox-LDL-) induced apoptosis in human aortic endothelial cells (HAECs). Following exposure to ox-LDL, HAECs presented with classical characteristics of apoptosis. However, EOFAZ ameliorated these morphological alterations and also inhibited the decrease in cell viability. In addition, EOFAZ abrogated the number of TUNEL or Hoechst 33258 stained positive cells observed after ox-LDL challenge. Investigation into the mechanisms of this inhibition revealed that EOFAZ treatment resulted in a downregulation of Bax and Caspase-3 at both the protein and mRNA expression levels. Moreover, EOFAZ was found to upregulate Bcl-2 protein and mRNA levels and to attenuate ox-LDL-induced HAECs injury caused by apoptosis, revealing both its therapeutic potential for endothelial cell injury protection and its clinical application for atherosclerosis.

## 1. Introduction

Atherosclerosis is the most common pathological cause of cardiovascular diseases and develops as a consequence of multiple risk factors. Of these factors, oxidized low density lipoprotein (ox-LDL) plays an important role in the initiation and progression of atherosclerosis and its complications [1]. Ox-LDL promotes the growth and migration of smooth muscle cells (SMC), monocytes/macrophages, and fibroblasts and also causes oxidative stress and cell injury in endothelial cells (ECs), which in turn facilitates formations of atherosclerotic plaque [2]. ECs form the lining of blood vessels and regulate the vascular integrity and homeostasis, while endothelial injury and dysfunction contribute to the key pathological progression of atherosclerosis [3].

Regular fluctuations in cell proliferation and cellular apoptosis help sustain the integrity of the endothelial structure and function. Increased apoptosis of ECs, SMCs, and immune cells has been observed in atheromatous regions, resulting in a disruption of the endothelium barrier and creating leaks that destroy the vascular wall integrity [4]. Ox-LDL induces vascular endothelial cell apoptosis via binding to and activating the lectin-like ox-LDL receptor 1 (LOX-1) [5]. Furthermore, mechanisms involved in ox-LDL-induced ECs apoptosis have been shown to be related to Fas receptors and Fas ligand (FasL) [6], activation of caspase-3 and caspase-9, downregulation of Bcl-2 expression, and activation of regulatory signal transducers, such as STAT [7] and NF-*κ*B [8].

*Zingiberaceae *belongs to the ginger family, consisting of a large number of aromatic perennial species known to have potential use in a number of cardiac-related therapies. For instance,* Alpinia zerumbet* (Pers.) Burtt et Smith is an aromatic plant originating in the East Indies widely exploited as local folk medicine in South America, Oceania, and Asia that contains several bioactive constituents and possesses broad spectrum pharmacological properties, such as antihypertensive, cardiovascular protective effects and antioxidant effects [9].

*Alpinia zerumbet* (called Yan shan jiang in China) is widely used as a local* miao* folk herb in Guizhou province. The roots, leaves, stems, and fruits of this plant have been applied to many different therapeutic uses [10], and the chemical constituents of each part are different [11]. Of note, the essential oil of EOAZ has attracted several investigations. We have recently demonstrated that the essential oil of Fructus* Alpiniae zerumbet* (EOFAZ) exerts protective effects in human umbilical vein endothelial cells (HUVECs) and ameliorates the oxidative stress induced by ox-LDL via elevating the activities of superoxide dismutase, catalase, and glutathione peroxidase, increasing the glutathione levels and attenuating the content of malondialdehyde [12]. Moreover, we later found that EOFAZ induces vasodilation through the nitric oxide/cyclic guanosine monophosphate (NOS-cGMP) pathway [13]. Taken together, we hypothesize that EOFAZ may possess a crucial protective effect in cardiovascular dysfunction. In the present study, we evaluate the protective effect of EOFAZ on ox-LDL-induced endothelial cell injury.

## 2. Methods and Materials

### 2.1. Chemicals and Herbs

The essential oil was extracted from the fruit of* Alpinia zerumbet*, collected in Zhenfeng county, Guizhou province, China, October 2009, according to methods previously described [13]. The fruit was identified by Professor Chen Zuyun and a voucher specimen (number 20091026) was deposited at the Research Division of Pharmacology, Guiyang Medical University (Guiyang, China). Briefly, chopped fruit was placed in a glass flask connected at one end to a glass vessel with water and at the other end to a water-cooled condenser. The water was heated to boiling point and the steam was percolated through the chopped fruit and collected in the condenser. After condensation, the watery phase containing the solutes (termed the “hydrolate”) was separated from the oily phase; the essential oil rediluted in water is termed the “pseudohydrolate.” The yield of essential oil was 1.3%.

Sixty-two compounds were separated and fifty-eight were identified from the essential oil. The composition of EOFAZ was determined by gas chromatography and mass spectrometry: *β*-phellandrene (16.388%), *β*-pinene (15.056%), 1,8-cineole (10.956%), camphene (10.120%), *α*-pinene (9.275%), linalool L (4.026%), camphor (3.657%), O-cymene (3.384%), *β*-myrcene (3.189%), borneol L (2.446%), caryophyllene oxide (1.778%), and terpinen-4-ol (1.756%); over 12 volatile compounds accounted for 82.03% of the total. EOFAZ was dissolved in dimethylsulfoxide (DMSO) at 10 mg/mL; control cells were therefore incubated in 0.04% (v/v) DMSO with equal volume of the highest dose of EOFAZ.

### 2.2. Materials

We purchased 3-(4,5-dimethylthiazol-2-yl)-2,5-diphenyltetra zolium bromide (MTT) from Sigma-Aldrich (St. Louis, MO). Ox-LDL was purchased from Yiyuan Biotechnology (Guangzhou, China). Terminal deoxynucleotidyl transferase- (TdT-) mediated dUTP Nick-End Labeling (TUNEL) apoptosis assay kit, Hoechst 33258 staining solution, lysis buffer, and bicinchoninic acid (BCA) assay kit were all obtained from Beyotime Institute of Biotechnology (Jiangsu, China). Bax (1 : 1000), Bcl-2 (1 : 1000), and *β*-actin (1 : 800) antibodies were purchased from Santa Cruz Biotechnology, Inc. (Santa Cruz, CA) and Caspase-3 (1 : 400) was purchased from Boster (Wuhan, China).

### 2.3. Cell Culture

Primary human aortic endothelial cells (HAECs) were obtained from Science Cell (San Diego, USA) and stored at Beijing Yuhengfeng biotech, Inc. (Beijing, China). HAECs were cultured in accordance with the manufacturer's protocol and incubated in a humidifier at 37°C with 5% CO_2_. HAECs at passages 3–5 were collected for use in all experiments.

### 2.4. MTT Assay

HAECs were seeded into 96-well plates (5 × 10^4^ cells/mL with 100 *μ*L), pretreated with EOFAZ (1, 2, or 4 *μ*g/mL, *n* = 5 for each concentration) for 2 h, and then exposed to 150 *μ*g/mL ox-LDL for 24 h. EOFAZ was freshly prepared as a stock solution in DMSO and diluted with culture medium. Following treatment we removed the supernatant and incubated the cells with 80 *μ*L medium and 20 *μ*L MTT (5 mg/mL) for 4 h and then lysed them with 100 *μ*L DMSO. Subsequently, spectrometric absorbance (570 nm) was measured using a microplate reader (Sunrise RC, Tecan, Switzerland). Survival rate (%) was calculated using the equation:

(1) $$\begin{array}{c} \text{Survival}\;\text{rate}\;\left(\%\right)=\frac{{\text{OD}}_{\text{treated}}}{{\text{OD}}_{\text{control}}}\times 100 \end{array}$$

### 2.5. TUNEL Assay

TUNEL assay was performed by using a one-step TUNEL apoptosis assay commercial kit, according to the manufacturer's protocol. Images were observed and captured with a fluorescence microscope (Olympus, Tokyo, Japan) (450–500 nm excitation) (515–565 nm emission) at 100× magnification. Five randomly chosen fields were analyzed for each group. The percentages of apoptotic cells were calculated from the ratio of TUNEL positive cells to total cells.

### 2.6. Hoechst 33258 Staining Assay

Cells were seeded on cover slides in 6-well plates (1 × 10^5^ cells/well), treated for 24 h, fixed with 4% paraformaldehyde for 20 min, and then incubated in Hoechst 33258 staining solution for 20 min at 37°C. The cells were analyzed with a fluorescence microscopic system (Olympus; 100× magnification, excitation wavelength 352 nm, emission wavelength 461 nm). Five randomly chosen fields were analyzed for each group. The rate of apoptotic cells was equal to the ratio of apoptotic cells to the number of total cells.

### 2.7. Western Blotting

Cells were collected and lysed in lysis buffer (Beyotime Institute of Biotechnology, Jiangsu, China). Lysates were clarified by centrifugation at 4°C for 15 min at 13,000 ×g and the concentration of protein in the supernatant was measured using a bicinchoninic acid (BCA) assay kit with a Varioskan multimode microplate spectrophotometer (Thermo Waltham, MA). Subsequently, proteins (30 *μ*g) were separated by 12% sodium dodecyl sulfate polyacrylamide gel electrophoresis (SDS-PAGE) and transferred to a PVDF membrane (Millipore, Bedford, MA). The membrane was blocked with 5% nonfat milk for 1 h, incubated with primary antibodies (listed above) for 1 h at 37°C and overnight at 4°C, rinsed in PBST, and finally incubated in secondary antibodies for 1 h at room temperature. Blots were visualized by using an enhanced chemiluminescence kit (Millipore, Billerica, MA). Digital images of blots were produced by a Syngene Gel Imaging System and quantified with GeneSnap (Syngene) software.

### 2.8. Reverse Transcription-PCR (RT-PCR)

Total RNA was isolated using a TRIzol kit (Invitrogen, Carlsbad, CA). Complementary DNAs (cDNAs) were synthesized using 500 ng of total RNA with Oligo-(dT) 18 primer at 42°C for 30 min and terminated by heating at 85°C for 5 min. Primers were synthesized by Sangon (Shanghai China, Table 1). PCR amplification was performed at 94°C for 5 min, 54°C for 30 seconds, and 72°C for 90 seconds with 35 cycles with a PCR Mastercycler (Eppendorf, Hamburg, Germany). The products were analyzed by Genesnap software on a 1.5% agarose gel containing 10 mg/mL ethidium bromide. Bax, bcl-2, and caspase-3 mRNA levels were quantified in relation to *β*-actin mRNA level.

### 2.9. Statistical Analysis

Statistical significance was calculated by one-way ANOVA and multiple comparisons were carried out by Dunnett's post hoc test (2-sided). A value of *P* < 0.05 was considered significant. Data are expressed as mean ± S.E.M. Each experiment was performed in triplicate.

## 3. Results

### 3.1. Protective Effect of EOFAZ on Cell Viability in Ox-LDL-Induced HAEC Injury

**Figure 1:**
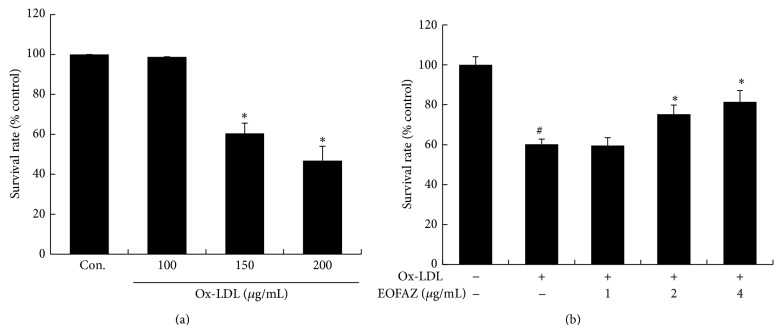
Cell viability detected by MTT assay. (a) Ox-LDL-induced cell injury in HAECs. (b) The protective effect of EOFAZ on cell viability. HAECs pretreated with EOFAZ (1, 2, or 4 *μ*g/mL) for 2 h prior to treatment with ox-LDL for 24 h. This treatment is used in the following experiments. The survival rates are shown by the mean ± S.E.M of three experiments: ^#^ *P* < 0.05, compared with control group; ^*^ *P* < 0.05, compared with ox-LDL group.

We measured the viability of HAECs utilizing MTT assays. Results show that ox-LDL causes a dose-dependent decrease in HAEC viability (Figure 1(a)). Both 150 and 200 *μ*g/mL of ox-LDL treatment for 24 h significantly reduced cell viability to 60.5% ± 5% and 46.8% ± 7% (control = 100%, *P* < 0.05), respectively; 150 *μ*g/mL ox-LDL was used for all subsequent experiments. Preincubation with EOFAZ at 2 *μ*g/mL and 4 *μ*g/mL significantly restored cell viability to 75.2% ± 5% and 81.4% ± 6%, as compared to ox-LDL alone (60.2% ± 3%) (Figure 1(b)), indicating that EOFAZ prevents ox-LDL-induced decreases in cell viability.

### 3.2. EOFAZ Ameliorates the Cell Morphology of Ox-LDL-Induced HAEC Injury

**Figure 2:**
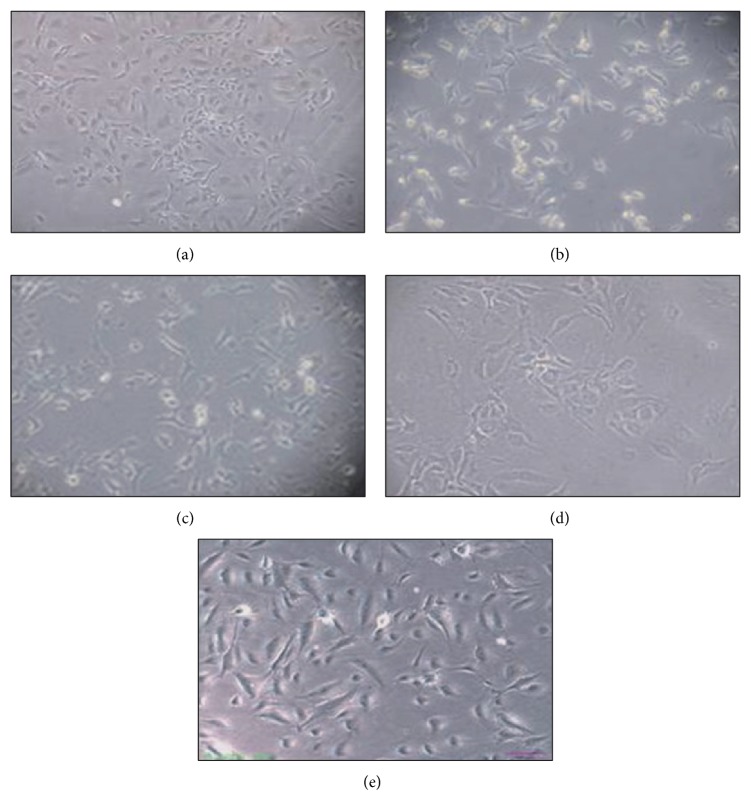
The protective effect of EOFAZ on cell morphology. (a) Control, (b) ox-LDL, (c) ox-LDL+EOFAZ (1 *μ*g/mL), (d) ox-LDL+EOFAZ (2 *μ*g/mL), and (e) ox-LDL+EOFAZ (4 *μ*g/mL). Representative images were captured by an inverted phase contrast microscope equipped with a color camera at 100× magnification.

Ox-LDL treatment of HAECs for 24 h resulted in the classical characteristics, displaying severely distorted physical traits such as irregular shapes and membrane shrinkage, and a number of cells also became round in shape (Figure 2(b)). Pretreatment with EOFAZ abrogated these morphological effects, especially at 4 *μ*g/mL (Figures 2(c)–2(e)). Compared with untreated cells (Figure 2(a)), EOFAZ (4 *μ*g/mL) exhibited no obvious differences in morphology, demonstrating healthy shapes and clear skeletons (Figure 2(e)). Taken together, these results show that EOFAZ restrains ox-LDL-induced cellular morphological changes.

### 3.3. EOFAZ Alleviated Ox-LDL-Induced Apoptosis in HAECs

**Figure 3:**
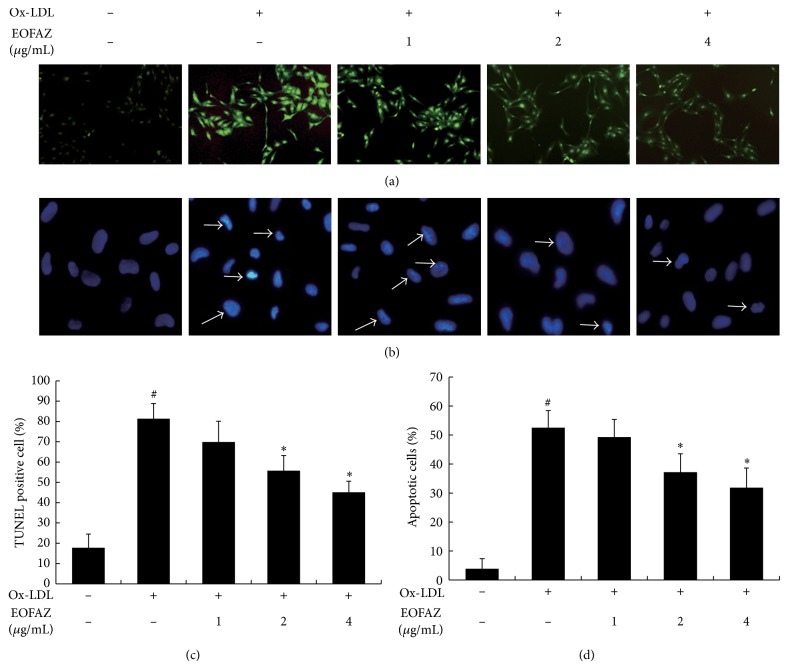
EOFAZ alleviated ox-LDL-induced apoptosis in HAECs. (a) TUNEL staining. Representative images (100× magnification) of cells stained with TUNEL (green fluorescence). (b) Hoechst 33258 staining. Representative images (100× magnification) of nuclei stained with Hoechst 33258 (blue fluorescence). The cells with typical apoptosis features are marked with arrows. (c) Quantitative analysis of TUNEL positive cells. (d) Quantitative analysis of apoptotic cells by Hoechst 33258 staining. Data are represented by mean ± S.E.M from three independent experiments. ^#^ *P* < 0.05, compared with control group; ^*^ *P* < 0.05, compared with ox-LDL group.

To determine whether EOFAZ ameliorated HAECs apoptosis induced by Ox-LDL we examined with TUNEL and Hoechst 33258 staining assays. As shown in Figure 3(a), the fluorescent intensity in the control group was low, while cells incubated with ox-LDL exhibit strong fluorescence intensity. Following analysis, we found that the percentage of TUNEL positive cells induced by ox-LDL increases from 17.8% ± 7% in control group to 81.4% ± 7%. Preincubation with EOFAZ (1, 2 and 4 *μ*g/mL) deceased the fluorescence intensity to 69.9% ± 10%, 55.7% ± 8%, and 45.1% ± 5%, respectively (Figure 3(c)).

We further evaluated the nucleolus changes in HAECs by Hoechst 33258 staining. Control cells presented with light fluorescence and the chromatin was equally distributed within the nucleolus (Figure 3(b)). Following ox-LDL treatment the cells displayed the typical features of apoptosis, including a congregation of the chromatin and nucleolus pyknosis, which emits a very bright fluorescence (Figure 3(b)). These features were not largely observed in the EOFAZ-pretreated cells. Quantification results show that ox-LDL significantly induces apoptosis in HAECs by 52.5% ± 6% while apoptosis was inhibited by up to 37.2% ± 6% and 31.8% ± 7% with 2 and 4 *μ*g/mL of EOFAZ treatment, respectively (Figure 3(d)). Taken together, these results clearly demonstrate that EOFAZ can suppress ox-LDL-induced apoptosis of HAECs in a concentration-dependent manner.

### 3.4. Relative Apoptotic Molecular Expression Levels

**Figure 4:**
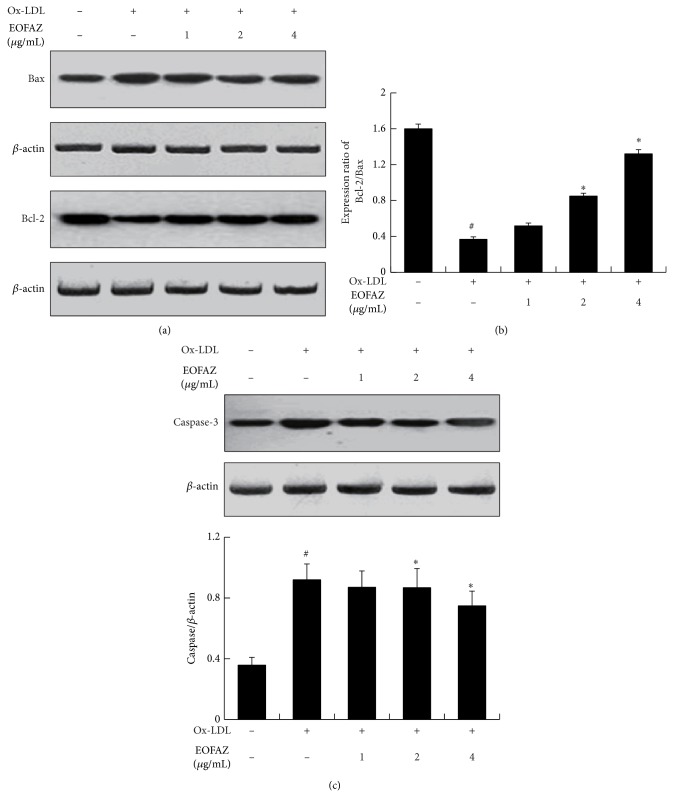
Western blot analysis of apoptosis related molecules expression following treatment with ox-LDL and EOFAZ. (a) Bcl-2 and Bax and densitometric analysis. (b) Caspase-3 and densitometric analysis. *β*-actin was used as the loading control. ^#^ *P* < 0.05, compared with control group; ^*^ *P* < 0.05, compared with ox-LDL group.

**Figure 5:**
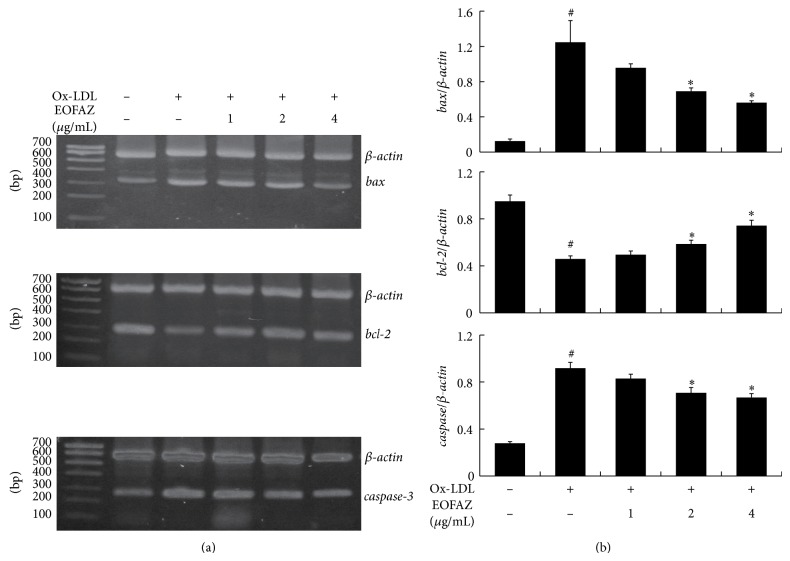
Effect of EOFAZ on* bax*,* bcl-2*, and* caspase-3* mRNA levels. (a) mRNA levels of* bax*,* bcl-2* and* caspase-3*. (b) Quantification of the mRNA level changes normalized to*β*-*actin*. ^#^ *P* < 0.05, compared with control group; ^*^ *P* < 0.05, compared with ox-LDL group.

We next performed western blots and RT-PCR to evaluate a few apoptosis-related molecules affected by EOFAZ. Western blots revealed that ox-LDL treatment causes an increase in the levels of Bax and caspase-3 and a concurrent decrease in Bcl-2. Following pretreatment with EOFAZ, the observed downregulation of Bcl-2 was inhibited, while the upregulation in Bax was reduced significantly (Figure 4(a)). Quantitative analysis showed that ox-LDL significantly decreases the ratio of Bcl-2 to Bax and that EOFAZ pretreatment abrogated this antiapoptotic index. In addition, ox-LDL-induced caspase-3 was also significantly inhibited by EOFAZ (Figure 4(b)). Similarly, RT-PCR results show that changes in mRNA levels of* bax*,* bcl-2*, and* caspase-3* are consistent with the changes in the protein levels following EOFAZ and ox-LDL treatment (Figures 5(a) and 5(b)). These results suggest that EOFAZ exerts antiapoptotic effects induced by ox-LDL via increasing* bcl-2* mRNA levels to upregulate the Bcl-2 content and via decreasing* bax* and* caspase-3* mRNA levels to downregulate their protein levels.

## 4. Discussion

Atherosclerosis is the primary cause of heart disease and stroke. Currently, despite some recent advances in atherosclerosis therapy, the primary therapeutic options (surgery and drug treatment) still have their drawbacks. Therefore, novel research into identifying effective agents and developing new therapeutic strategies for the treatment of this deadly disease is necessary [14]. The fruit of the* Alpiniae zerumbet* is widely used against cardiovascular disease in Guizhou province, China. Through the years, our lab has reported that the EOFAZ possesses significant medicinal effects on anti-inflammation and antihypertension and on protection against cardiovascular diseases [12, 13, 15, 16]. As such, we strongly believe that EOFAZ is a promising drug for atherosclerosis treatment.

Dysfunction of the vascular endothelium is recognized as a primary event in the pathogenesis of atherosclerosis and is a critical target for preventing or slowing the progression of this disease [17]. In addition, the study of human endothelium has become a central part of cardiovascular research. In the present study, we used HAECs to investigate the protection effect of EOFAZ in atherosclerosis. Abnormalities of lipid metabolism flow lead to the formation of atherosclerotic lesions [18]. Certain post secretory modifications in the structure of lipoproteins affect their atherogenic potential. Oxidation of lipoproteins such as ox-LDL is one of the strongest mechanisms of atherogenesis and is known to induce endothelial cell apoptosis, ultimately leading to endothelial injury and loss of the vascular wall integrity [19]. At present, we also found ox-LDL to significantly decrease cell viability and to alter the cellular morphology. However, these effects were inhibited by EOFAZ pretreatment. Furthermore, we previously found that EOFAZ can resist ox-LDL-induced oxidative stress [12]. As such, in the present study, we also set out to determine whether EOFAZ can suppress ox-LDL-induced apoptosis and clearly found that pretreatment with EOFAZ was able to abrogate the apoptotic effects of ox-LDL on HAECs.

We next wanted to determine a mechanism for the antiapoptotic effects of EOFAZ. Bcl-2 family proteins are known to play an important role in the regulation of apoptosis. Among them, the antiapoptotic protein Bcl-2 acts as a repressor of apoptosis, whereas the proapoptotic protein Bax acts as a promoter; the ratio of Bcl-2 to Bax is directly correlated with apoptosis [20]. Ox-LDL has been found to induce apoptosis by decreasing the cellular levels of Bcl-2 [21] and by increasing Bax in human coronary cells [22] and HUVECs [23]. Similarly, we also found that ox-LDL increases the protein and mRNA levels of Bax, while downregulating Bcl-2 in HAECs. Interestingly, EOZAF markedly inhibited this alteration in the Bcl-2/Bax ratio, indicating that the protective effect of EOZAF occurs by affecting this pathway at some molecular level. Caspase activation is a hallmark of apoptosis induction [24] and is considered as an antiatherosclerotic drug target during endothelial apoptosis and atherosclerosis [25]. EOFAZ also attenuated the ox-LDL-mediated increase in caspase-3 mRNA and protein levels.

Apoptosis is a complex biological process involving many pathways (primarily the death receptor pathway and the mitochondrial pathway) that ultimately converge at caspase-3 [26]. The mitochondrial pathway utilizes the mitochondria and the Bcl-2 family of proteins; the ratio of Bcl-2/Bax is crucial for the activation of the mitochondrial apoptotic pathway. It has also been shown that caspase-9 and caspase-3, but not caspase-8, play roles in the ox-LDL-induced apoptotic signaling pathway [27]. Consistent with these results, we found that both the ratios of Bax/Bcl-2 and caspase-3 were increased in cells treated with ox-LDL, indicating that the intrinsic apoptotic pathway is involved in ox-LDL-induced apoptosis in HAECs. Moreover, EOFAZ pretreatment resulted in a decrease in caspase-3 and Bax expression, but an increase in Bcl-2 expression, suggesting that EOFAZ impedes ox-LDL induced apoptosis by interfering with the mitochondrial pathway. Ox-LDL activates and upregulates its own receptor LOX-1 to induce apoptosis in ECs and is associated with reactive oxidative species (ROS) generation, release of cytochrome c, NF-*κ*B transcriptional activation, and subsequent modulation of the expression of some apoptotic genes, such as* caspase-3* and* bcl-2*. Whether EOFAZ affects the LOX-1-ROS-NF-*κ*B signaling pathway to cause the observed inhibition of endothelial apoptosis still needs our further investigation. In previous work, we separated 62 compounds, and 58 of them were identified to be derived from EOFAZ. Therefore, the compounds responsible for the antiapoptosis activity of HAECs induced by ox-LDL remain to be resolved. Moreover, we need to explain whether these components act on ox-LDL itself or work to break ox-LDL.

## 5. Conclusion

EOFAZ attenuates ox-LDL-induced endothelial cell injury by interfering with cellular apoptosis through a mechanism involving the apoptotic factors, Bax, Bcl-2, and caspase-3. This study sheds light on the application of EOFAZ for the treatment of cardiovascular diseases. Additional studies should be carried out to isolate and evaluate the active constituents of EOFAZ responsible for this effect. Moreover, the exact molecular mechanisms involved in the protective effect of EOFAZ on ox-LDL-induced endothelial cells injury remain to be resolved in future studies.

## Supplementary Material

Table S1: Chemical constituents in essential oil and their relevant contents of Fructus *Alpiniae Zerumbet*.
Figure S1: The relevant contents of 16 identified main compounds in EOFAZ. GC-MS chromatograms of EOFAZ.

## Figures and Tables

**Table 1:** Primer sequences.

Gene	Sequence	Product (bp)
*bax *	Sense: 5′ GTTGTCGCCCTTTTCTACTT 3′	316 bp
	Antisense: 5′ AAAACGCATTATAGACCACATC 3′	

*bcl-2 *	Sense: 5′ GCAGAGGGGCTACGAGTGG 3′	304 bp
	Antisense: 5′ GGTGAAGGGCGTCAGGTGC 3′	

*caspase-3 *	Sense: 5′ CAGAACTGGACTGTGGCATT 3′	295 bp
	Antisense: 5′ CATGGAATCTGTTTCTTTGC 3′	

*β*-*actin *	Sense: 5′ GGCCGGGACCTGACTGACTA 3′	580 bp
	Antisense: 5′ AGCATTTGCGGTGGACGAT 3′	
