# Supplementary material for: Essential Oils from Fructus A. zerumbet Protect Human Aortic Endothelial Cells from Apoptosis Induced by Ox-LDL In Vitro
Source: Evid Based Complement Alternat Med. 2014 Dec 23;2014:956824. doi: 10.1155/2014/956824 (PMC4290151; doi:10.1155/2014/956824)
Supplement: Supplementary file 1 — Table S1: Chemical constituents in essential oil and their relevant contents of Fructus Alpiniae Zerumbet. Figure S1: The relevant contents of 16 identified main compounds in EOFAZ. GC-MS chromatograms of EOFAZ. [file 956824.f1.docx]

**Table S1** Chemical constituents in essential oil and their relevant contents of Fructus *Alpiniae Zerumbet*.

| No. | RT | Compound | | Molecular Formula | | Molecular Weight | RC (%) | |  |
| --- | --- | --- | --- | --- | --- | --- | --- | --- | --- |
|  | 3.98 | | Isobutyl acetate | | C_6_H_12_O_2_ | 116 | | 0.211 | |
|  | 4.49 | | Hexanal | | C_6_H_12_O | 100 | | 0.041 | |
|  | 5.97 | | Ethylbenzene | | C_8_H_10_ | 106 | | 0.011 | |
|  | 6.38 | | Isoamyl acetate | | C_7_H_14_O_2_ | 130 | | 0.450 | |
|  | 6.45 | | 2-Methylbutyl acetate | | C_7_H_14_O_2_ | 130 | | 1.157 | |
|  | 7.61 | | Tricyalene | | C_10_H_16_ | 136 | | 0.473 | |
|  | 7.79 | | α-Thujene | | C_10_H_16_ | 136 | | 0.884 | |
|  | 8.06 | | α-pinene | | C_10_H_16_ | 136 | | 9.275 | |
|  | 8.50 | | Camphene | | C_10_H_16_ | 136 | | 10.120 | |
|  | 8.81 | | Benzaldehyde | | C_7_H_6_O | 106 | | 0.032 | |
|  | 9.38 | | β- pinene | | C_10_H_16_ | 136 | | 15.056 | |
|  | 9.74 | | β-Myrcene | | C_10_H_16_ | 136 | | 3.189 | |
|  | 9.99 | | α-Terpipene | | C_10_H_16_ | 136 | | 0.313 | |
|  | 10.12 | | α-Phellandrene | | C_10_H_16_ | 136 | | 1.525 | |
|  | 10.28 | | δ-3-Carene | | C_10_H_16_ | 136 | | 0.297 | |
|  | 10.81 | | O-Cymene | | C_10_H_16_ | 136 | | 3.384 | |
|  | 10.97 | | β-Phellandrene | | C_10_H_16_ | 136 | | 16.388 | |
|  | 11.10 | | 1,8-Cineole | | C_10_H_18_O | 154 | | 10.956 | |
|  | 11.47 | | (Z)-Ocimene | | C_10_H_16_ | 136 | | 0.218 | |
|  | 11.79 | | γ-Terpinene | | C_10_H_16_ | 136 | | 0.842 | |
|  | 12.20 | | (Z)-Linalool oxide | | C_10_H_18_O_2_ | 170 | | 0.025 | |
|  | 12.66 | | α-Terpinolene | | C_10_H_16_ | 136 | | 0.435 | |
|  | 13.13 | | Linalool L | | C_10_H_18_O | 154 | | 4.026 | |
|  | 13.51 | | Fenchol | | C_10_H_18_O | 154 | | 0.109 | |
|  | 13.73 | | Stereoisomer-p-menth-2-en-1-ol | | C_10_H_18_O | 154 | | 0.198 | |
|  | 14.28 | | 1-Terpinene | | C_10_H_18_O | 154 | | 0.150 | |
|  | 14.40 | | Camphor | | C_10_H_16_ O | 152 | | 3.657 | |
|  | 14.52 | | Exo-methyl-camphenilol | | C_10_H_18_O | 154 | | 0.366 | |
|  | 15.09 | | Borneol L | | C_10_H_18_O | 154 | | 2.446 | |
|  | 15.40 | | Terpinen-4-ol | | C_10_H_18_O | 154 | | 1.756 | |
|  | 15.62 | | Cryptone | | C_9_H_14_O | 138 | | 0.496 | |
|  | 15.80 | | α-Terpineol | | C_10_H_18_O | 154 | | 1.199 | |
|  | 17.15 | | Cuminal | | C_10_H_12_O | 148 | | 0.090 | |
|  | 17.25 | | Benzylacetone | | C_10_H_12_O | 148 | | 0.071 | |
|  | 17.54 | | Pineritone | | C_10_H_16_O | 152 | | 0.017 | |
|  | 18.13 | | Phellandral | | C_10_H_16_O | 152 | | 0.123 | |
|  | 18.38 | | Bormyl acetate | | C_10_H_16_O | 152 | | 0.273 | |
|  | 19.07 | | Carvacrol | | C_12_H_20_O_2_ | 196 | | 0.081 | |
|  | 20.46 | | Neryl acetate | | C_12_H_20_O_2_ | 196 | | 0.030 | |
|  | 20.84 | | α-Copaene | | C_15_H_24_ | 204 | | 0.033 | |
|  | 21.26 | | β-Elemene | | C_15_H_24_ | 204 | | 0.038 | |
|  | 22.01 | | β-Caryophllene | | C_10_H_14_O | 150 | | 1.233 | |
|  | 22.87 | | α-Humulene | | C_15_H_24_ | 204 | | 0.267 | |
|  | 23.84 | | γ-Cadinene | | C_15_H_24_ | 204 | | 0.031 | |
|  | 23.91 | | β-Selinene | | C_15_H_24_ | 204 | | 0.023 | |
|  | 24.00 | | α-Muurolene | | C_15_H_24_ | 204 | | 0.054 | |
|  | 24.36 | | α-Amorphene | | C_15_H_24_ | 204 | | 0.266 | |
|  | 24.56 | | δ-Cadinene | | C_15_H_24_ | 204 | | 0.213 | |
|  | 25.51 | | Farnesol | | C_15_H_26_O_2_ | 222 | | 0.706 | |
|  | 26.07 | | Caryophyllene oxide | | C_15_H_24_O | 220 | | 1.778 | |
|  | 26.66 | | Humulene epoxide Ⅱ | | C_15_H_24_O | 220 | | 0.148 | |
|  | 27.18 | | γ-Eudesmol | | C_15_H_26_O_2_ | 222 | | 0.144 | |
|  | 27.62 | | β-Eudesmol | | C_15_H_26_O_2_ | 222 | | 0.128 | |
|  | 27.69 | | α-Eudesmol | | C_15_H_26_O_2_ | 222 | | 0.188 | |
|  | 34.11 | | Palmitic acid | | C_16_H_32_O_2_ | 256 | | 0.430 | |
|  | 37.38 | | Linoleic acid | | C_18_H_32_O_2_ | 280 | | 0.455 | |
|  | 37.48 | | Oleic acid | | C_18_H_34_O_2_ | 282 | | 1.496 | |
|  | 37.82 | | Stearic acid | | C_18_H_36_O_2_ | 284 | | 0.947 | |


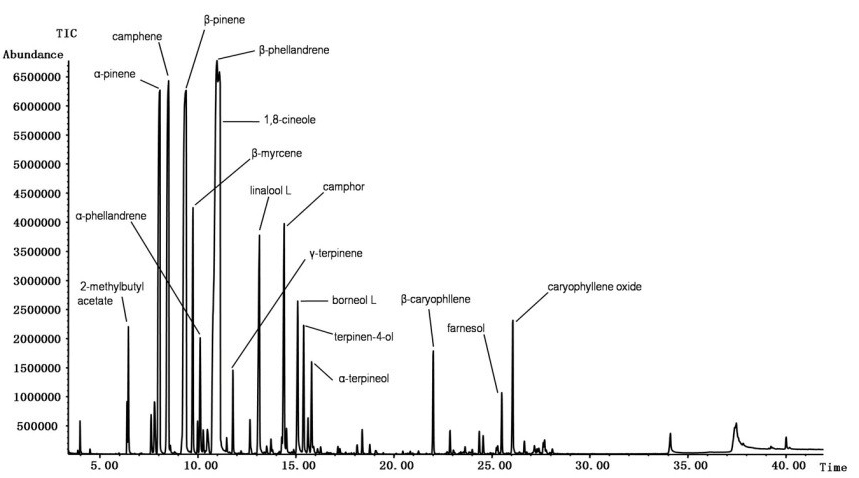


**Figure S1.** The relevant contents of 16 identified main compounds in EOFAZ. GC-MS chromatograms of EOFAZ.
